# Low awareness and common misconceptions about schistosomiasis in endemic lowland areas in Western Ethiopia: a mixed-methods study

**DOI:** 10.1186/s12889-021-11106-y

**Published:** 2021-06-04

**Authors:** Alemayehu Assefa, Berhanu Erko, Svein Gunnar Gundersen, Girmay Medhin, Nega Berhe

**Affiliations:** 1 University Of Assosa, College Of Health Science, P.O. Box 18, Assosa, Ethiopia; 2 Akililu Lemma Institute of Pathobiology, P.O. Box 1176, Addis Ababa University, Addis Ababa, Ethiopia; 3 Department of Global Development and Planning, University of Agder, Oslo, Norway

**Keywords:** Knowledge, Attitude, Practices, Mass drug administration, Sustainable control, Schistosomiasis, Ethiopia

## Abstract

**Background:**

Understanding the health behavior of the target population is crucial for sustainable schistosomiasis control. The aim of this study was to assess schistosomiasis related levels of knowledge, attitude, and practices of communities in lowland areas of western Ethiopia, where schistosomiasis is endemic.

**Methods:**

A community-based multilevel triangulation mixed-methods design was conducted in three schistosomiasis endemic villages in the Abbey and Didessa valleys of the Benishangul Gumuz Region of Western Ethiopia, where mass drug administration (MDA) was done 30 years back and again the last 5 years. A structured survey questionnaire, in-depth interviews, focused group discussions, and observation was conducted to assess levels of knowledge, attitude, and practices related to schistosomiasis in the communities.

**Results:**

Among the survey participants, 13% reported having heard of schistosomiasis, locally called Pecka (meaning worm). The majority of this 13% believe that schistosomiasis is caused by the biting of the worm Pecka, while others say drinking dirty water is the cause of infection, or they didn’t know what the cause is. A majority of respondents answered “I don’t know” to most of the questions about established knowledge of schistosomiasis. Male participants and students were more aware of schistosomiasis than their counterparts, and awareness increased with the educational level. Only one participant perceived that schistosomiasis was a serious disease. There were negative attitudes and misconceptions about the drug used in the mass treatment and many complaints were raised related to the size of the tablet and its side effects. There was no local budget and specific plan to prevent and control the disease. Local health personnel had insufficient knowledge about schistosomiasis, and the diagnosis and treatment capacities of local health institutions were poor.

**Conclusion:**

In the current research area, schistosomiasis prevention and control recommendations should be redesigned to change the knowledge, attitudes, and practices of the community and local health workers. It is also necessary to have the local budget and trained manpower in order to diagnose and treat schistosomiasis locally. There is a great need to have a safer Praziquantel pediatric formulation.

**Supplementary Information:**

The online version contains supplementary material available at 10.1186/s12889-021-11106-y.

## Operational definitions

For the purpose of the current study we used the following definitions:

Knowledge: Awareness or familiarity of the participants with schistosomiasis, its control and preventions.

Attitude: The manner in which participants view schistosomiasis and how this view affects the way they might view the need for managing risky behaviors, and an ongoing intervention program (MDA).

Practice: The habitual way of the participants’ response to schistosomiasis, its prevention and an ongoing intervention program (MDA).

## Introduction

Despite the global control efforts of schistosomiasis, the prevalence, and worm loads remain high, particularly in Sub-Saharan Africa (SSA), which accounts for about 90% of people living with schistosomiasis [1]. Of the five known species of *Schistosoma*, the most prevalent in SSA are *S. haematobium* which causes urogenital schistosomiasis, and *S. mansoni* which causes intestinal schistosomiasis [2].

The distribution and impacts of *Schistosoma* in endemic countries in SSA differ significantly, with a greater impact on the poor and marginalized communities [3]. These populations are living in areas that have a low socioeconomic status with limited access to clean water and adequate sanitation. In most of the schistosomiasis endemic countries, people who have frequent contact with infected natural water bodies, because of the nature of their work are at a greater risk of infection [4]. The high *Schistosoma* infection risk among these groups is mainly due to risky water contact practices, poor sanitation, and lack of knowledge, and misconceptions about schistosomiasis [5].

Community awareness about parasitic infections and a better understanding of the socio-cultural and behavioral determinants of the targeted community affect the magnitude and prevention of the infections and additionally support in designing effective prevention and control strategies [6, 7]. The active participation of the targeted communities in the prevention and control efforts is one of the key ways for the success and sustainability of disease prevention and control programs [8]. Likewise, understanding the perception of the targeted population is of great importance for developing evidence-based, and cost-effective intervention plans [9].

In communities where poverty and schistosomiasis are a double burden, intervention through public awareness is often recommended as the first line and cost-effective action to create the enabling environment for other strategies to succeed [10]. The World Health Organization (WHO) inspires endemic countries to adopt policies and programs focusing on an integrated approach of Mass Drug Administration (MDA) programs with primary health care interventions, especially, with health education programs, which help in eliminating schistosomiasis rather than just controlling its morbidity [11].

The finding of a recent systematic review and meta-analysis in Ethiopia showed that the prevalence of schistosomiasis is high despite continued prevention and control efforts [12]. Although the knowledge, attitude, and practice (KAP) of the community could have a significant role in the success of any control strategies, such studies towards schistosomiasis are limited in the country. Hence, this study was aimed to investigate the levels of schistosomiasis related to KAP of schistosomiasis endemic community in the remote lowlands of the Abbey and Didessa Valleys in Benishangul Gumuz Region of Western Ethiopia, during an ongoing MDA campaign and 30 years after the first schistosomiasis control was attempted.

## Methods

### Study area and population

The details of the study area are presented elsewhere [13]. The area is located in the remote lowlands of western Ethiopia with poor infrastructure. Benishangul Gumuz Region is one of the nine regional states of Ethiopia which shares a border with Sudan from the west, Amhara regional state from the East, and Orommia regional state from the south. The Grand Ethiopian Renaissance Dam (GERD) is being constructed on the Nile River in this regional state. The current study was conducted in three of the same villages of the region, where Gundersen et al. [14] observed a lasting effect of campaigns and mass deworming 20–30 years previously. The villages were: (a) Chessega village in the Sirba Abbey area of the Abbey Valley and (b) in the other two villages, Metti and Shimala in the adjacent Agallu Metti area, situated eastwards on the hilly slopes south of the junction between the Didessa and Abbey rivers (700-1200 m altitudes). The area is an extensions of the Sudanese savannah with a hot, dry climate and receives seasonal rains from May to October.

The study area is about 600 km west of Addis Ababa and mostly inhabited by a Nilotic ethnic group known as Gumuz, except some Oromos and Amharas who moved to the lowland for farming or public services. The total population of the three study villages is about 8375, of which 2660 are residents of Sirba Abbey (Chessega), 3800 are residents of Metti, and 1915 are residents of Shimala (Agallu Metti). All the inhabitants live under similar poor environmental sanitation and low socio-economic status. They earn their living in small-scale farming using the traditional farming methods. Maize and millet are the most commonly cultivated crops in the area. *Schistosoma mansoni* is known to be an endemic disease, but the status of this endemic has been unknown for the last 20 years.

### Study design

We used the multilevel triangulation-mixed methods design, involving individuals, community groups, and health care providers and program implementers.

### Sample size determination

This study was nested within another community-based study that examined the prevalence of schistosomiasis for which sample size was determined [13] using 63% as the best available estimate for the prevalence of *S.mansoni* reported in a previous school-based cross-sectional study in the same area [14]. The sample size and households that were used for that study [13] were used to address the objective stipulated in this paper. For the qualitative data, the planned starting number was 12 FGDs, 6 with men and 6 with women, and 25 in-depth interviews. The final numbers of FGDs and in-depth interviews conducted were decided based on information saturation.

### Survey sampling procedure

Three villages were purposely selected based on schistosomiasis endemicity after consultation with the District Public Health Officials and previous studies [14]. The total sample size was distributed to the three villages proportional to their population size. Using the list of households obtained from the Kebele administration in each village, we used a systematic sampling technique to select households for interviews. From each household, an individual whose age was at least 12 years old, who lived in the study area for more than a year and not having any obvious critical medical illness at the time of data collection were eligible for the study provided they give consent/assent to be part of the study. In case the study participant was younger, there should be an adult, parents or guardians, in the household who could give written informed consent on behalf of the child.

### Survey questionnaire

The survey questionnaires were prepared in English and translated to Amharic and back to English to check the content change. The questionnaire had three sections: The first part covered demographic data and the second part focused on the knowledge and level of awareness of the respondents regarding schistosomiasis. Questions like a local name for schistosomiasis, sources of information about the disease, signs, and symptoms, methods of transmission, and prevention were included in the 2nd part of the questionnaire. The third part covered the perceived severity of the disease and the attitudes towards interventions. The respondents were asked about their perception of the severity and preventability of the disease and the medical intervention provided by the government as well as any comments regarding the interventions.

### Qualitative data

#### In-depth interviews

Purposive sampling technique was used to select key informants (KIs) for in-depth interviews. The KIs include district administrators, teachers, religious or clan leaders and district health officials, and health care providers. The in-depth interview guide covered topic areas that helped us to explore the insights of the KI’s knowledge, attitude, and practices about schistosomiasis infection, its prevention, and control.

#### Focus group discussions

Focus Group Discussions (FGDs) were done with men and women community members in each village. The FGDs were led by trained moderators and note-takers fluent in the local languages. The first author developed themes and sub-themes on the subject of discussion which was then used to probe the discussants. An evaluation was done at the end of each FGD to validate the collected data. The whole discussions were audio reordered, transcribed verbatim, translated into English, coded, and then analyzed using thematic analysis.

### Observation

We conducted observations to assess schistosomiasis risk behaviors in the community, including the availability and proper use of toilet facilities, water contact activities, and open defecation behaviors. We also observed the availability of diagnostic material for schistosomiasis in local health institutions, the availability of schistosomiasis medications, case registration logs of schistosomiasis cases, and schistosomiasis management guidelines.

### Quality assurance

Quality assurance measures included training data collectors, field testing of the survey instruments with a special focus on a ‘real-life’ situation to improve the process and to enhance the understanding of the field data collection team. Field supervisors and the first author were involved in the fieldwork to immediately review questionnaires on a daily basis and to correct any inconsistencies that may arise. Three Ph.D. candidates, two as data collectors and one supervisor were trained for 3 days by an experienced qualitative researcher and by the first author of this paper. The training was included understanding the aim of the study, a common understanding of survey questionnaires and interview guides, ethical issues and consenting procedures, session management and moderating, probing, and data collection procedures. Data cleaning was a multi-stage process. After entering the data in Epidata, the data was cleaned immediately, and it was continuously exported into SPSS for preliminary analysis of quantitative data. Data cleaning was also continued during and after translation, transcription, and coding of qualitative data. Transcripts were reviewed by the first author at each site for translation accuracy and revised when necessary.

### Data management and analysis

Quantitative data were entered into the Epidata software. Statistical analysis was done using IBM SPSS version 25 software. Descriptive statistics including frequencies and proportions were used to summarize the data. Chi-square test and Fisher’s exact tests were used to test the significance of associations between variables and a *P*-value below 5% was considered as an indicator of statistical significance.

The qualitative data were analyzed manually using the thematic method [15]. All the qualitative data collected were tape-recorded, and verbatim responses to each question were translated and transcribed, and documented in Microsoft Word. We checked the consistency of the transcripts against the audio files to ensure the accuracy of the transcribed files. A code sheet was created following the focus group and the key informant guides after which, the textual data was coded into selected themes and a master sheet analysis was carried out, giving all the responses from the focused group discussions and key informants interview. Thematic analysis was used where responses were categorized into themes and then ideas were formulated by looking at the patterns of responses. Analyzed data were presented in text form. The qualitative data were analyzed by three Ph. D candidates. The analysis team discussed and resolved the differences in coding or themes, revised the codebook or themes accordingly, and re-coded as necessary to ensure consistent application of code.

## Results

### Quantitative results

#### Socio-demographic characteristics

A total of 376 households (i.e. one person from one household whose age was at least 12 years of age) were interviewed. Table 1 shows socio-demographic data, also indicating the educational status and occupation of the participants.

**Table 1:** Socio-demographic characteristics of community participants (*N* = 376)

Socio-demographic profiles	Numbers (376)	Percent
Sex		
Male	199	52.9
Female	177	47.1
Age categories		
12–19	200	53.2
20–27	114	30.3
28–35	56	14.9
36^+^	6	1.6
Ethnic group		
Gumuz	312	83.0
Oromo	52	13.8
Shenasha	4	1.1
Amhara	6	1.6
Others	2	0.5
Educational status of participants		
Primary	222	59.0
Secondary	58	15.5
Collage and above	34	9.0
Not formally educated	62	16.5
Participant Occupation		
Farmer	103	27.4
Student	225	59.8
Salaried employee	38	10.1
Merchant	10	2.7

#### Awareness and knowledge about schistosomiasis

Only 12.8% (48/376) of the participants heard about schistosomiasis and the main sources of information were family or friends (45.8%) and schools (36.7%). The local name thought for schistosomiasis was Pecka (meaning biting worm living in dirty water) (Table 2).

**Table 2:** Awareness and knowledge of study participants about schistosomiasis

Knowledge areas	Responses	Number	Percent
Know anything about schistosomiasis/ Bilarziasis (*n* = 376)	Yes	48	12.8
	No	328	87.2
Sources of information for those aware of schistosomiasis (*n* = 48)	Health facilities	10	20.4
	School	18	36.7
	Family/friends	22	45.8
Local name for schistosomiasis (among schistosomiasis aware participants) (*n* = 48)	Pecka (snail worm)	31	64.6
	Bilharzias	14	29.2
	Schistosomiasis	3	6.3
Societal etiologies/causes of schistosomiasis (among schistosomiasis aware participants) (*n* = 48)	Pecka biting	30	62.5
	Drinking dirty water	4	8.3
	Eating food longtime after cooked	3	6.3
	I don’t know	11	22.9
Knows ways of schistosomiasis infection (among schistosomiasis aware participants) (*n* = 48)	Yes	35	72.9
	No	13	27.1
Mentioned ways of infections (among those aware infection methods) (*n* = 35)	Contact with Pecka /snails	22	62.9
	Drinking contaminated water	8	22.9
	While fishing	1	2.9
	While swimming/bathing	31	88.6
	While fetching water	2	4.3
Knows symptoms of schistosomiasis (among schistosomiasis aware participants) (*n* = 48)	Yes	19	39.6
	No	29	60.4
If yes, what are they? More than one response was possible (among those aware symptoms) (*n* = 19)	Blood in stool	1	5.3
	Abdominal pain	8	42.1
	Swollen abdomen	1	5.3
	Itching	19	100.0
Knows prevention methods (among schistosomiasis aware participants) (*n* = 48)	Yes	9	18.8
	No	39	81.2
Mentioned prevention methods (among those aware prevention methods) (*n* = 9)	Avoiding contact with water bodies	6	66.7
	Treatment/MDA	7	77.8

Among those who ever heard of schistosomiasis, 62.5% (30/48) said that the cause for schistosomiasis is Pecka worm biting, whereas 22.9% (11/48) responded that they did not know the cause. Of those who were aware of schistosomiasis, 73% (35/48) said that they knew the ways of schistosomiasis transmissions: 88.6% (31/35) said while swimming/bathing in water bodies, 62.9% (22/35) said contact with worm and 22.9% (8/35) said drinking contaminated water. Among those who said they were aware of schistosomiasis, 39.6% (19/48) or 5% of all study participants said that they knew the symptoms of schistosomiasis and all of them mentioned itching as the most common symptom. Among those who said they were aware of schistosomiasis, 18.8% (9/48) indicated that they knew the prevention methods. Of this group of respondents, 77.8% (7/9) said treatment/MDA and 66.7% (6/9) said avoiding contact with water bodies that contain Pecka worm (Table 2). None of the study participants had participated in health education about schistosomiasis during the last 12 months.

#### Factors that affect knowledge of people about schistosomiasis

Awareness of schistosomiasis was significantly associated with sex (*x*^*2*^ = 7.08, *P* = 0.008), village (*x*^*2*^ = 10.41, *P* = 0.006), education level (*x*^*2*^ = 8.94, *P* = 0.030), and occupation (F = 7.80, *P* = 0.023). There was no significance difference in awareness about schistosomiasis among various age groups (F = 2.11, *P* = 0.665), whereas, awareness about mass treatment of schistosomiasis was significantly different across age groups (F = 85.61, *P* < 0.001), village (*x*^*2*^ = 69.30, *P* < 0.001), educational level (*x*^*2*^ = 84.16**,** *P* < 0.001), and occupation (F = 93.50, *P* < 0.001) (Table 3).

**Table 3:** Schistosomiasis related awareness and associated factors

Background characteristics		Aware of schistosomiasis		*P*-value	Aware of schistosomiasis MDA		*P*-value
		Yes Number (%)	No Number (%)		Yes Number (%)	No Number (%)	
Sex	Male	34(17.1)	165(82.9)	0.008	50(25.1)	149(74.9)	0.611
	Female	14(7.9)	163(92.1)		59(33.3)	118(66.7)	
Age group	12–19	27(56.3)	173(52.7)	0.665	97(48.5)	103(51.5)	< 0.001
	20–27	12(10.5)	102(89.5)		10(8.8)	104(91.2)	
	28–35	8(14.3)	48(85.7)		2(3.6)	54(96.4)	
	36+	1(16.7)	5(83.3)		0	6(100)	
Villages	Chessega	25(20.8)	95(79.2)	0.006	27(22.5)	93(77.5)	< 0.001
	Metti	16(9.5)	153(90.5)		82(48.5)	87(51.5)	
	Shimala	7(8.0)	80(92.0)		0	87(100)	
Educational status	Primary	28(12.6)	194(87.4)	0.030	104(46.8*)*	118(53.2)	< 0.001
	Secondary	11(19.0)	47(81.0)		2(3.4)	56(96.4)	
	Collage+	7(20.6)	27(79.4)		2(5.9)	32(94.1)	
	Not educated	2(3.2)	60(96.8)		1(1.6)	61(98.4)	
Occupation	Farmer	8(7.8)	95(92.2)	0.245	3(2.9)	100(97.1)	< 0.001
	Student	31(13.8)	194(86.2)		104(46.2)	121(53.8)	
	Employee	8(21.1)	30(78.9)		2(5.3)	36(94.7)	
	Merchant	1(10.0)	9(90.0)		0	10(100)	

#### Perception and attitudes on susceptibility, severity, and medical intervention (MDA)

From the total of 48 participants who said that they were aware of schistosomiasis, only 22.9% (11/48) agree that they were at the risk of *Schistosoma* infection during contact with river or stream water bodies. Only one individual said that schistosomiasis is a serious disease, whereas the majority 93.7% (45/48) said that they do not know (Table 4).

**Table 4:** Perceptions and attitudes of study participants on schistosomiasis and intervention programs

Perception/ attitudes areas	Responses	Number	Percent
At risk of infection by *Schistosoma (n = 48)*	Agree	11	22.9
	Disagree	1	2.1
	I don’t know	36	75.0
At risk of infection during swimming/bathing/washing/fetching (*n* = 48)	Agree	11	22.9
	I don’t know	37	77.1
Schistosomiasis is a serious disease (*n* = 48)	Agree	1	2.1
	Disagree	2	4.2
	I don’t know	45	93.7
Schistosomiasis is preventable (*n* = 48)	Agree	5	10.4
	Disagree	1	2.1
	I don’t know	42	87.5
Faces/ urine is the primary source of infection (*n* = 48)	Disagree	4	8.3
	I don’t know	44	91.7
Knows MDA for schistosomiasis (*n* = 376)	Yes	109	29.0
	No	267	71.0
Everybody should dewormed (*n* = 109)	Agree	73	67.0
	Disagree	6	5.5
	I don’t know	30	27.5
MDA is effective in treatment/controlling schistosomiasis (*n* = 109)	Agree	3	2.8
	Disagree	1	0.9
	I don’t know	105	96.3
A drug given as MDA tests bad (*n* = 109)	Agree	99	90.8
	Disagree	1	0.9
	I don’t know	9	8.3
A drug given as MDA may cause serious side effects (*n* = 109)	Agree	101	92.7
	I don’t know	8	7.3
Dewormed during the last schistosomiasis deworming campaign (*n* = 376)	Yes	84	22.3
	No	292	77.7
Reasons for none deworming (*n* = 292)	Fearing of side effects	13	4.4
	Providers not come to our home	260	95.6
	I think it is not important	4	1.4
	Absent during the campaign	4	1.4
	It tastes bad	6	2.1
	I am not eligible	3	1.0

Only 10.4% (5/48) of those who said that they were aware of schistosomiasis agree that schistosomiasis is preventable. None of the participants agree that human excreta are a primary source of schistosomiasis infection. From a total of the study participants, 29% (109/376) participants said that they were aware of schistosomiasis MDA. Of this group of respondents, 67% (73/109) agree with its importance, and 2.8% (3/109) agree that the mass treatment is effective for schistosomiasis treatment or control. Among those who said they were aware of schistosomiasis MDA, 90.8% (99/109) agree that the tablet for mass treatment was tasted badly and 92.7% (101/109) thought that the tablet causes serious side effects (Table 4).

#### Schistosomiasis prevention and control practices: prevention and treatments

The majority of the study participants said that they cross water bodies on the way to school or work and 38.6% had gone to a river or stream at least once a day to swim/bath or fetch water. The main sources of water for bathing were rivers and streams. From our observation during fieldwork, we confirmed that; washing, swimming, and bathing in rivers, and streams were common practices irrespective of age and sex. We also observed that the rivers are very close to the villages that even under five children spend most of their daytime in the rivers and streams.

The main source of drinking water for the majority of the communities (81.4%) was a public hand pump. River and stream were the main sources of drinking for those households who do not have access to the public hand pumps, and for all the community members when they were out of their homes for farming or other activities. It was also confirmed through observation during the fieldwork.

A great majority (87.5%) said that they have latrine facilities. Although only 28.9% reported regular use of the latrine, and 3.6% reported that, latrines are not in use. Field observations showed that most of the latrines were extremely filthy, had no doors, shared between many families, and almost collapsed making most of the latrines not convenient for use. We also confirmed open defecation was a common practice. Rivers and streamsides were full of human excreta.

### Qualitative results

#### Socio-demographic characteristics of participants (focus group discussions (FGDs) and in-depth interviews)

A total of 64 individuals participated in 20 in-depth interviews and 6 FGDs were held in the three villages. Males and females were equally represented in FGDs, but the number of males was larger than females during in-depth interviews. The youngest participant was a 22 year-old and the oldest was a 65 year-old community leader. Seventy-seven percent of females and 68% of male participants involved in FGD did not have formal education. All women who participated in FGD were housewives, whereas men participants were farmers.

#### Program based plans and activities on schistosomiasis at the district health office

There was a position named neglected tropical diseases (NTDs) officer. Their role was coordinating the mass drug administration (MDA) that is conducted once a year. Except for MDA, these officials did not have other specific plans for the prevention and control of schistosomiasis. A senior official said*:* “*We do not have strategic plans related to prevention and control of schistosomiasis, but only MDA campaign which is conducted on a yearly basis. This is so because we do not have a budget allocated for such activities.”* Another senior official said: *“Schistosomiasis is not a serious health issue in the area, it is not common currently, because of the MDA campaign, so, it is not needed to allocate a budget for the prevention of this disease.”*

None of the health officials mentioned schistosomiasis as a common public health problem in the area and they did not know the local name of the disease*.* These officials did not believe that schistosomiasis could be controlled. For instance, senior health personnel said: *“I do not believe that schistosomiasis could be prevented and controlled, because of* ***l****ow awareness, and negative attitude of the community towards MDA, that they live near water bodies and pass most of their time in the water by fishing and swimming.”*

#### The capacity of district health facility towards schistosomiasis prevention and control

To assess the capacity of local health facilities, we conducted two key informant interviews at each health center. We also cross-checked the interviews with observation. A laboratory technician said: “*I did not take any training, but I could diagnose bilharzia by making stool seamier on a slide using normal saline and looking under a microscope for the Schistosoma eggs*.” This is a standard procedure to screen for schistosomiasis, although Kato thick smear technique is needed for egg quantification for which there were no tools available.

The health professionals working at the outpatient department (OPD) in the local health facilities did not have adequate knowledge on schistosomiasis. One of them said: “*I don’t know more about schistosomiasis. I heard that it is a parasitic disease that transmitted when a person drinks dirty stagnant water and when he eats contaminated foods*. *I think it is treated with a single pill of Praziquantel or Albendazole*.” By reviewing 1 year case registration documents at OPD, we did not find any schistosomiasis cases registered or reported at the two health centers, although, there were Praziquantel drugs available at the stores.

This all indicates that schistosomiasis was not diagnosed and treated in the local health facilities, due to a lack of material and skill.

#### Awareness and knowledge about schistosomiasis

The great majority of the participants involved in in-depth interviews and focused group discussions said that they heard about schistosomiasis, which was locally called “Pecka”. A 65 year-old community leader said*:* “*Yes, I know bilharzia, and we call it Pecka, it is a worm living in the water attached to stones that bite on bare parts of our body, and it causes severe itching.”*

The majority of the participants did not mention schistosomiasis as a common public health problem in the area, although few teachers and religious leaders reported itching as a common challenge among students. A teacher from a primary school said: *“Malaria is a common reason for the absences of students from schooling and itching is a common challenge for students to concentrate and attend the class.”* And, a 45 year-old religious leader said*:* “*… during religious gatherings, I have seen most people scratching their body.”*

Most of the participants had inadequate information and poor awareness of transmission methods, symptoms, and prevention methods of schistosomiasis. None of the participants mentioned that open defecation was the main source of infection. A 35 year-old uneducated housewife said: “*We only know that bilharzia is caused by a worm biting from water. We do not know if it is due to open defecation. Defecating in the bush on the sides of the rivers is common.”*

The majority of the participants involved in in-depth interviews and FGDs heard about schistosomiasis MDA provided in the area, and their source of information was their school children. However, they did not have adequate information. A primary school teacher said*: “We simply support them when they come to school to provide the pills, they said it is for Bilarziasis, but neither teachers nor students received training about, and we do not know about the pill more in depth.”* A 65 year-old community leader said*: “sometimes I heard that the doctors were giving the treatment at school, they did not come to our home.”*

Some community members indicated that they did not hear about schistosomiasis MDA that was being provided in the area despite 5 years of schistosomiasis MDA campaign in the area. A primary school teacher said: *“It is you, whom I have seen the first time who come to school to talk about Bilarziasis in my experience. I do not know anything about the bilharzia MDA campaign you talk about, but sometimes the health personnel come and gave pill for students, but I am not sure for what propose it was*.” A 45 year-old religious leader said: “*I do not know the pills for bilharzia, but I heard of a pill that has been given for onchocerciasis.”*

#### Attitude and perceptions about schistosomiasis

Most of the participants involved in in-depth interviews and FGD perceives that they were at risk for *Schistosoma* infection, but none of them knew the right mechanisms of transmission. All who heard about bilharzia believe that it was due to biting of worm while barefoot and stepping on the worms, which lives on stones in stagnating dirty water. A 35 year-old Kebele leader said: *“Bilharzia is transmitted by Pecka a worm that lives in dirty stagnant water attached to stones. Since, the biting is when standing on worms, other forms of water contact: swimming, bathing, and crossing without standing on worms have no problem*.”

The majority of the participants perceive bilharzia as a simple short time problem, commonly itching and abdominal pain. A 65 year-old community leader said*:* “*I am living my whole life here, so, I do not think it is a major problem. Our major problem is malaria…”.*

The majority of the participants had a negative attitude towards the Praziquantel tablet. The majority believe that the drug causes serious health problems. A 40 year-old farmer said: *“…*. *it causes severe vomiting and abdominal burning when children take it. Most of the children who see the event do not want to take it again.”* The side effects were described by a few participants as being more severe than the disease itself. A 36 year-old farmer said: “*That drug is dangerous. My child was not able to move for days. I was feeling shocked at the time. Thanks to God finally he survived.”* A village leader said*:* “… … .*the community is also complaining about this drug at a meeting by reporting that their children are sick. Really the issue is challenging in our area*.” Senior official said: *“...the acceptance is generally low, for example, some community members believe that the tablet is a birth controlling pill, to make them infertile and reduces the numbers of the ethnic group.”* Especially, the acceptance of the MDA was very low among adolescents. A primary school teacher said: *“To tell the truth most of the students do not want to take the pill, especially those students above grade five are not voluntary. They also tell the younger students as the drug is dangerous*.”

The majority of the participants did not believe that Bilarziasis is preventable and could be controlled. A senior official said*: “We do not believe we could prevent and control schistosomiasis. Because, of low awareness, living habits, and negative attitude of the communities towards MDA.”* And, a farmer said*: “How could we prevent the worm from biting? We do not have traditional or modern medicine.”*

#### Comments on schistosomiasis preventions and control from the community

The participants recommended the following as the strategy to increase the acceptance of the mass treatment by the community: giving the tablets at home to care for their children and monitoring the side effects of the pills, making the pills smaller and safer, giving the pills to the whole community, giving health education on the disease for the community, and training local health personnel and strengthen the local health system and having a local budget for the prevention and control of the disease. A 25 years-old housewife said: *“I think the problem with the tablets is due to the fact that children took the drug having an empty stomach at school without having a reasonable amount of food. Why not they are allowed to take to home and take the drug after eating. We think it is good if it is done that way.”* A religious leader said*: “It’s good if we could get information on transmission methods, and prevention methods if possible.”* A teacher at one primary school said: *“The personnel from the health office should teach the community and also should work with the schools.”* An officer in the local health office said*: “Strengthening the health system and having a local budget for prevention and control of the diseases is important.”*

## Discussion

The present study indicates limited knowledge, common negative attitudes, and risky practices related to schistosomiasis in the general population, health staff, and officials. It is obvious that any information by educational campaigns during the first treatment campaign 30 years ago has not been kept up. Moreover, the presently ongoing MDA does not seem to involve the local inhabitants, teachers, health- and other officials to the extent that they have obtained proper schistosomiasis knowledge.

The local health officials did not have specific plans for prevention and control of schistosomiasis except for the MDA campaigns organized by the upper level of the health care system. This finding is similar to a report in Burundi, where knowledge of care providers for intestinal schistosomiasis was very low [16], and in Ghana, where very low knowledge of genital schistosomiasis by health workers was reported [17]. However, lower knowledge level than report in Senegal [18]. This difference may be due to differences in the level of education and having specific training on schistosomiasis. None of the local health care providers in our area were specifically trained on schistosomiasis, unlike that of Senegal’s report. The results of our study showed that material resources for clinical and laboratory diagnosis of schistosomiasis were lacking at the local health facilities and they were not diagnosing and treating schistosomiasis locally. Such shortages were also reported in a study from Burundi [16].

A few surveyed community participants said that they had heard about schistosomiasis and only a very few of them indicated at least one transmission method, symptom, and prevention method. Although most of the participants involved in in-depth interviews and FGDs had heard about schistosomiasis, only a few had detailed knowledge. Contrary to our observations, previous studies in endemic areas of Ethiopia found a high level of awareness of schistosomiasis [19–21]. Likewise, our present observation of KAP about schistosomiasis is lower than studies from other endemic countries: in Yemen [22, 23], in Kenya [24–26], in South Africa [6], in Uganda [27], in Nigeria [28], and in Mozambique [29]. This difference may be due to the geographical periphery of the area and that the large majority of the communities belong to a Nilotic group who were less educated and the rest few were Oromo’s and Amhara’s from highland for farming, who are also poor, less educated farmers and daily laborers.

We had observed that many misconceptions exist, especially, regarding disease transmission and prevention. A majority of participants were believing that schistosomiasis is caused by the Pecka worms biting when people stand on them barefooted. This misconception was also reported in a study in Kenya [30]. And some believe schistosomiasis is transmitted by drinking dirty water. This misconception was also reported by most studies from different endemic areas: in Ethiopia [19], in Yemen [22], and in South Africa [31]. This misconception promotes risky behaviors unless corrective actions have been taken.

Few of those who indicated that they were aware of schistosomiasis, said they knew the prevention methods, of which some said treatment/MDA and others said not walking barefooted in the water bodies which contain the worm. Whereas the majority said they do not know the prevention methods. Poor knowledge about prevention methods of schistosomiasis is also reported in a study from Mozambique [29], which may indicate a lack of integrated schistosomiasis control and elimination plans in most developing endemic countries.

The results of this study revealed that males were more aware of schistosomiasis than females. This observation is similar to studies from different endemic areas [6, 21, 22, 31]. This may be due to in developing endemic countries, females are less educated than males and culturally less participation of females in public meetings and gatherings. Although more participants from Chessega had heard about schistosomiasis, participants from Metti had detailed knowledge on transmission, symptoms, and privation therapy. None of the participants from Shimala were aware of schistosomiasis MDA. This might be due to better advocacy and coverage of MDA in Metti since Metti is a center for the Districts.

The percentage of participants who heard about schistosomiasis increased with the educational level, but there was no difference in detailed knowledge. Primary school students were more aware of MDA, probably due to the ongoing school campaigns. These observations are contrary to a study in Mozambique, which reported better knowledge of risky behaviors, prevention methods, and treatments among higher educated [29]. We found no schistosomiasis awareness difference among age groups, while a study in Kenya reported an increase in awareness on schistosomiasis transmission with age [25].

Only a few participants agree that they are at the risk of *Schistosoma* infection. Only one participant indicated schistosomiasis as a serious disease. This finding is different from a study in Kenya, where everyone was perceived to be at the risk of schistosomiasis infection and perceived the disease as very serious [30]. This may be due to poor case identification and poor awareness in our study area. None of the respondents agree that human excreta are a primary source of infection for the disease schistosomiasis. This finding is unlike a Kenyan study where the participants’ perceived open defecation as a cause of schistosomiasis [30]. This is another indicator of poor awareness of the community, and poor health information communications in the area.

Only a very few participants agree that the MDA is effective in curing or control of the disease schistosomiasis. We observed, there were highly negative attitudes towards the praziquantel drug which is used for mass treatment. The most majority believe that the pill is tasting bad and it had serious side effects. Berhe et al. have described this from Ethiopia already in 2009 [32]. They found that most abdominal side effects could be prevented by giving the tablet with a small meal, and information that the abdominal cramps would be only transitional. Similar findings are indicated in a study from Uganda [33]. The side effects were described by a few participants as being more severe than the disease itself. There was also a misconception considering the pill as birth control pills. These perceptions will continuously affect the uptake of MDA [33] unless corrective information is available to the community.

Even though there were some public hand pumps, the main sources of water for the community were rivers and streams. Our data is indicating that; washing, swimming, and bathing in rivers and streams were common practices, irrespective of age and sex in the community. Open-air defecation was very common, rivers and streamsides are full of human excreta. A study shows that bathing after urination or defecation near rivers and streams facilitates schistosomiasis transmission [34]. These practices are also, a critical obstacle to schistosomiasis prevention and control efforts.

## Conclusion

In the present study areas, the awareness about schistosomiasis was very low, even among the local health personnel. There was also common misconception on transmissions and negative attitudes towards the ongoing mass treatment and common complain about the size and safety of the tablet. There was no local budget and specific local plan for the prevention and control of schistosomiasis. Hence, if the disease prevention and control are ultimate to be achieved, the following strategic plans should be needed nationally and locally: first**,** strengthening the local health system (having a local budget and trained manpower), advocacy, coordination, and partnerships; secondly**,** including community stakeholders in developing and delivering awareness programs, which enhance the efficiency of community engagements and information dissemination channels. Thirdly, it required to include developmental interventions such as safe water provision to minimize exposure to the infected water bodies and livelihood activities predisposing the community to infection. Fourthly enhancing neglected tropical diseases monitoring and evaluation, surveillance, and research in such neglected areas. Finally, incorporating strategies that address misconceptions and myths on disease into health awareness activities and control measures. We also, like to recommend international concerned bodies to give due attention to safer Praziquantel pediatrics formulation.

## Supplementary Information

**Additional file 1: Supplementary file 1** In-depth interviews guide.pdf

**Additional file 2: Supplementary file 2** Focused Group Discussions (FGD) guide.pdf

**Additional file 3: Supplementary file 3** Schistosoma infection risky behaviors observation guide.pdf

**Additional file 4: Supplementary file 4** Data.xlsx

**Additional file 5: Supplementary file 5** Authors’ information.pdf

**Additional file 6: Related file 1** Reference lists.pdf

## Data Availability

The quantitative data used to draw a conclusion for the present study is available in the manuscript, whereas the qualitative data including the audio records and transcripts of the interviews, are not publicly available due to concerns regarding confidentiality and data protection but are available from the corresponding author on reasonable request.

## Abbreviations

FGDs: Focus group discussions
GERD: Great Ethiopian renaissance dame
KAP: Knowledge, attitude and practice
MDA: Mass drug administration
NTDs: Neglected tropical diseases
SSA: Sub- Saharan Africa
STHs: Soil transmitted helminths
WHO: World health organization
