# Supplementary material for: Low awareness and common misconceptions about schistosomiasis in endemic lowland areas in Western Ethiopia: a mixed-methods study
Source: BMC Public Health. 2021 Jun 4;21:1064. doi: 10.1186/s12889-021-11106-y (PMC8178865; doi:10.1186/s12889-021-11106-y)
Supplement: Supplementary file 1 — Additional file 1: Supplementary file 1 In-depth interviews guide.pdf [file 12889_2021_11106_MOESM1_ESM.pdf]

Low awareness and common misconceptions about schistosomiasis in endemic lowland areas in western Ethiopia. A mixed-methods study.

1. *In-depth interview with clan or religious leaders*

Socio-Demographic Profile

1. *Date of interview:* ____________
2. *Cod:* ________________________
3. *Settlement Woreda/district:* _____________________________________
4. *Settlement Village:* ____
5. *Participant's ? Gender Male [ ] Female [ ]*
6. *Participant's ? Age (in years):* _______
7. *Participant's level of education? None [ ] Primary [ ] Secondary [ ] collage and above [ ]*
8. *Participant's occupation? Farming [ ] Salaried worker [ ] student [] Merchant [] Other, Specify* ________
9. *Health information on schistosomiasis/bilharzias in the last 12 months? Yes [] No []*

In-depth interview guide

1. What are the common health problems in your area? Could you explain?
2. Do you ever heard schistosomiasis? If yes, explain
    a. Its local name?
    b. Societal cause for schistosomiasis?
3. Is schistosomiasis is a common health problem in your area? Could you explain, why or why not?
4. Could you explain how schistosomiasis is transmitted to human? What are the risky behaviors?
5. Could you explain the sign -symptoms of schistosomiasis?
6. Is schistosomiasis is a treatable and preventable disease? How?
7. Is health office in your area giving attention to this disease?
    a. If yes, what they are doing to prevent?
    b. If no, why?
8. Is there schistosomiasis deworming program in your areas?
    a. If yes, how often?
    b. What is the response of the community toward the MDA?
9. Does individuals infected by this disease sick medical care:
    a. If yes, where?
    b. If no, why?
10. Is there any local means of preventing/treating this disease? If, yes explain?
11. Any comments you have on prevention and control of this disease?
12. Did you ever visited health facilities for treatment of schistosomiasis?
    a. If yes, how was the care provided to you by the health facilities?

b. If no, why not?

Thanks! For your time and giving me this valuable information.

2. <u>In-depth interview with Keble leaders</u>

Socio-Demographic Profile

10. Date of interview: ____________

11. Cod: ________________________

12. Settlement Woreda/district: _____________________________________

13. Settlement Village: ____

14. Participant's ? Gender Male [ ] Female [ ]

15. Participant's ? Age (in years): _______

16. Participant's level of education? None [ ] Primary [ ] Secondary [ ] collage and above [ ]

17. Participant's occupation? Farming [ ] Salaried worker [ ] student [] Merchant [] Other, Specify ________

18. Health information on schistosomiasis/bilharzias in the last 12 months? Yes [] No []

In-depth interview guide

13. What are the common health problems in your area? Could you explain?
14. Do you ever heard schistosomiasis? If yes, explain
    a. Its local name?
    b. Societal cause for schistosomiasis?
15. Is schistosomiasis is a common health problem in your area? Could you explain, why or why not?
16. Could you explain how schistosomiasis is transmitted to human? What are the risky behaviors?
17. Could you explain the sign -symptoms of schistosomiasis?
18. Is schistosomiasis is a treatable and preventable disease? How?
19. Is health office in your area giving attention to this disease?
    a. If yes, what they are doing to prevent?
    b. If no, why?
20. Is there schistosomiasis deworming program in your areas?
    a. If yes, how often?
    b. What is the response of the community toward the MDA?
21. Does individuals infected by this disease sick medical care:
    a. If yes, where?
    b. If no, why?
22. Is there any local means of preventing/treating this disease? If, yes explain?
23. Any comments you have on prevention and control of this disease?
24. Did you ever visited health facilities for treatment of schistosomiasis?
    a. If yes, how was the care provided to you by the health facilities?

b. If no, why not?

Thanks! For your time and giving me this valuable information.

3. ***<u>In-depth interview with teachers</u>***

Socio-Demographic Profile

1. *Date of interview: ____________*
2. *Cod: ________________________*
3. *Settlement Woreda/district: _____________________________________*
4. *Settlement Village: ____*
5. *Participant's ? Gender Male [ ] Female [ ]*
6. *Participant's ? Age (in years): _______*
7. *Participant's level of education? None [ ] Primary [ ] Secondary [ ] collage and above [ ]*
8. *Participant's field of study?_____________________________*
9. *Participant's years of services?(in yeras):____________________*
10. *Health information on schistosomiasis/bilharzias in the last 12 months? Yes [] No []*

In-depth interview guide

1. What are the common health problems among the students? Could you explain?
2. Do you know schistosomiasis? If yes, explain
    a. Its local name?
    b. Societal cause for schistosomiasis?
3. Is schistosomiasis is a common health problem in your area? Could you explain, why or why not?
4. Could you explain how schistosomiasis is transmitted to human? What are the risky behaviors?
5. Could you explain the sign -symptoms of schistosomiasis?
6. Is schistosomiasis is a treatable and preventable disease? How?
7. Is health office in your area giving attention to this disease?
    a. If yes, what they are doing to prevent?
8. Is there schistosomiasis deworming program in your areas/schools?
    a. If yes, how often?
    b. If no, why?
    c. If yes, what is the response of the students toward the MDA?
9. Does individuals infected by this disease sick medical care:
    a. If yes, where?
    b. If no, why?
10. Are schools giving attention to this disease?

a. If yes, what they are ding to prevent?

b. If no, why?

11. Any comments you have on prevention and control of this disease?
12. Did you ever visited health facilities for treatment of schistosomiasis?
    a. If yes, how was the care provided to you by the health facilities?
    b. If no, why not?

Thanks! For your time and giving me this valuable information.

4. *<u>In-depth interview with local health professionals at HP/HC/Hospitals/</u>*

Socio-Demographic Profile

1. *Date of interview: ____________*
2. *Cod: ________________________*
3. *Settlement Woreda/district: _____________________________________*
4. *Settlement Village: ____*
5. *Name of Health facility: ____________________________*
6. *Participant's ? Gender Male [ ] Female [ ]*
7. *Participant's ? Age (in years): _______*
8. *Participant's level of education? None [ ] Primary [ ] Secondary [ ] collage and above [ ]*
9. *Participant's Profession?_____________________________*
10. *Participant's years of services?(in yeras):____________________*
11. *Health information on schistosomiasis/bilharzias in the last 12 months? Yes [] No []*

In-depth interview guide

1. What are the common health problems in your area? Could you explain?
2. Do you know schistosomiasis? If yes, explain
    a. Its local name?
    b. It societal etiology?
    c. Scientific etiology?
3. Is schistosomiasis is a common health problem in your area? Could you explain, why or why not?
4. Could you explain how schistosomiasis is transmitted to human? What are the risky behaviors?
5. Could you explain the sign -symptoms of schistosomiasis?
6. Is schistosomiasis is a treatable and preventable disease? How?
7. Is health facilities in your area giving attention to this disease?
    a. If yes, what they are doing to prevent?
    b. If no, why?
8. Is there schistosomiasis deworming program in your areas?
    a. If yes, how often?

b. If no, why?
c. What is the response and perceptions of the community toward the MDA?

9. Does individuals infected by this disease sick medical care:
   a. If yes, how do you Dx and Rx them?
   b. What advising do you provide them after treatment?
   c. If no, why?
10. Do you have the following if patents with schistosomiasis come to your health facilities:
    a. Sufficient knowledge/skill to investigate and treat them?
    b. Laboratory for investigation?
    c. Medication for treatment?
11. Schistosomiasis is still very common in the area, what is the reasons you think for?
    a. Community related?
    b. Socio-geographic related?
    c. Health care system related?
    d. Schistosomiasis related?
12. Any comments you have on prevention and control of this disease?

Thanks! For your time and giving me this valuable information.

6. <u>In-depth interview with woreda health officials</u>

1. *Date of interview:* ____________
2. *Cod:* ________________________
3. *Settlement Woreda/district:* _____________________________________
4. *Settlement Village:* ____
5. *Name of Health facility:* ____________________________
6. *Participant's ? Gender Male [ ] Female [ ]*
7. *Participant's ? Age (in years):* _______
8. *Participant's level of education? None [ ] Primary [ ] Secondary [ ] collage and above [ ]*
9. *Participant's Profession?*_____________________________
10. *Participant's years of services?(in yeras):*____________________
11. *Health information on schistosomiasis/bilharzias in the last 12 months?   Yes []   No []*

In-depth interview guide

1. What are the common health problems in your area? Could you explain?
2. Do you know schistosomiasis? If yes, explain
   a. Its local name?
   b. It societal etiology?
   c. Scientific etiology?
3. Is schistosomiasis is a common health problem in your area? Could you explain, why or why not?
4. Could you explain how schistosoma is transmitted to human? What are the risky behaviors?
5. Could you explain the sign -symptoms of schistosomiasis?
6. Is schistosomiasis is a treatable and preventable disease? How?
7. Is schistosomiasis is one of your priority police area in health care?
   a. If yes, what you are doing to prevent?
   b. If no, why?
8. Is there deworming program in your areas?
   a. If yes, how often?

    b. If no, why?
    c. What is the response of the community to ward MDA?
9. Does individuals infected by this disease sick medical care:
    a. If yes, do you have trained human power, laboratory and medication to give effective care?
    b. If no, why?
10. What is the coverage/ availability of the following in your woreda:
    a. Safe drinking water? Common source of drinking water?
    b. Number of households having latrine? How functional?
    c. Deworming coverage?
11. Schistosomiasis is still very common in the area, what is the reasons you think for?
    a. Community related?
    b. Socio-geographic related?
    c. Health care system related?
    d. Schistosomiasis related?
12. Any comments you have on prevention and control of this disease?

Thanks! For your time and giving me this valuable information.
