# Supplementary material for: Low awareness and common misconceptions about schistosomiasis in endemic lowland areas in Western Ethiopia: a mixed-methods study
Source: BMC Public Health. 2021 Jun 4;21:1064. doi: 10.1186/s12889-021-11106-y (PMC8178865; doi:10.1186/s12889-021-11106-y)
Supplement: Supplementary file 2 — Additional file 2: Supplementary file 2 Focused Group Discussions (FGD) guide.pdf [file 12889_2021_11106_MOESM2_ESM.pdf]

Low awareness and common misconceptions about schistosomiasis in endemic lowland areas in western Ethiopia. A mixed-methods study.

1. ***<u>Focused Group Discussions (FGDs) with men community members</u>***

Socio-Demographic Profile

1. *Date of interview: ____________*
2. *Cod: ________________________*
3. *Settlement Woreda/district: _____________________________________*
4. *Settlement Village: ____*
5. *Participant's ? Gender Male [ ] Female [ ]*
6. *Participant's ? Age (in years): _______*
7. *Participant's level of education? None [ ] Primary [ ] Secondary [ ] collage and above [ ]*
8. *Participant's occupation? Farming [ ] Salaried worker [ ] Merchant [] Other, Specify ________*
9. *Health information on schistosomiasis/bilharzias in the last 12 months? Yes [] No []*

In-depth interview guide

1. What are the common health problems in your area? Could you explain?
2. Do you ever hear schistosomiasis? If yes, explain
    a. Its local name?
    b. Societal cause for schistosomiasis?
3. Have schistosomiasis been a common health problem in your area? Could you explain, why or why not?
4. Could you explain how schistosomiasis is transmitted to human? What are the risky behaviors?
5. Could you explain the sign -symptoms of schistosomiasis?
6. Is schistosomiasis is a treatable and preventable disease? How?
7. Is the health office in your area, giving attention to this disease?
    a. If yes, what they are doing to prevent?
    b. If no, why?
8. Is there schistosomiasis deworming program in your areas?
    a. If yes, how often?
    b. What is the response of the community toward the MDA?
9. Does individuals infected by this disease sick medical care:
    a. If yes, where? b. If no, why?
10. Is there any local means of preventing/treating this disease? If, yes, explain?
11. Any comments you have prevention and control of this disease?
12. Did you ever visit health facilities for treatment of schistosomiasis?
    a. If yes, how was the care provided to you by the health facilities?
    b. If no, why not?

Thanks! For your time and giving me this valuable information.

2. ***<u>Focused Group Discussions (FGDs) with women community members</u>***

Socio-Demographic Profile

10. *Date of interview: ____________*
11. *Cod: ________________________*
12. *Settlement Woreda/district: _____________________________________*
13. *Settlement Village: ____*
14. *Participant's ? Gender Male [ ] Female [ ]*
15. *Participant's ? Age (in years): _______*
16. *Participant's level of education? None [ ] Primary [ ] Secondary [ ] collage and above [ ]*
17. *Participant's occupation? Housewife [ ] Salaried worker [ ] Merchant [] Other, Specify ________*
18. *Health information on schistosomiasis/bilharzias in the last 12 months? Yes [] No []*

In-depth interview guide

13. What are the common health problems in your area? Could you explain?
14. Do you ever hear schistosomiasis? If yes, explain
    a. Its local name?
    b. Societal cause for schistosomiasis?
15. Have schistosomiasis been a common health problem in your area? Could you explain, why or why not?
16. Could you explain how schistosomiasis is transmitted to human? What are the risky behaviors?
17. Could you explain the sign -symptoms of schistosomiasis?
18. Is schistosomiasis is a treatable and preventable disease? How?
19. Is the health office in your area, giving attention to this disease?
    a. If yes, what they are doing to prevent?
    b. If no, why?
20. Is there schistosomiasis deworming program in your areas?
    a. If yes, how often?
    b. What is the response of the community toward the MDA?
21. Does individuals infected by this disease sick medical care:
    a. If yes, where? b. If no, why?
22. Is there any local means of preventing/treating this disease? If, yes, explain?
23. Any comments you have prevention and control of this disease?
24. Did you ever visit health facilities for treatment of schistosomiasis?
    a. If yes, how was the care provided to you by the health facilities?
    b. If no, why not?

Thanks! For your time and giving me this valuable information.
