# Supplementary material for: Low awareness and common misconceptions about schistosomiasis in endemic lowland areas in Western Ethiopia: a mixed-methods study
Source: BMC Public Health. 2021 Jun 4;21:1064. doi: 10.1186/s12889-021-11106-y (PMC8178865; doi:10.1186/s12889-021-11106-y)
Supplement: Supplementary file 3 — Additional file 3: Supplementary file 3 Schistosoma infection risky behaviors observation guide.pdf [file 12889_2021_11106_MOESM3_ESM.pdf]

Low awareness and common misconceptions about schistosomiasis in endemic lowland areas in western Ethiopia. A mixed-methods study.

Observations guide

1. Schistosoma infection risky behaviors and practices observation guideline a. Use of latrine vs open filed defecation

b. Availability and functionality of latrines

c. Contact habits with water bodies

d. Source of drinking water

a. Kato-Katz materials and other possible methods?

b. Praziquantel?

c. Registered schistosomiasis cases?

d. Guidelines on schistosomiasis diagnosis, treatment and prevention?

Name of woreda: ___________________________

Name of village: ___________________________

2. Availability of schistosomiasis diagnosing and treating materials in health facilities

Name of woreda: ___________________________

Name of village: ___________________________

Name of health facility:_____________________
