# Supplementary material for: Low awareness and common misconceptions about schistosomiasis in endemic lowland areas in Western Ethiopia: a mixed-methods study
Source: BMC Public Health. 2021 Jun 4;21:1064. doi: 10.1186/s12889-021-11106-y (PMC8178865; doi:10.1186/s12889-021-11106-y)
Supplement: Supplementary file 5 — Additional file 5: Supplementary file 5 Authors’ information.pdf [file 12889_2021_11106_MOESM5_ESM.pdf]

Authors' information

1. Berhanu Erko Roro: berhanue@yahoo.com
2. Svein Gunnar Gundersen: s.g.gundersen@gmail.com
3. Girmay Medhin Tesfay: gtmedhin@yahoo.com
4. Nega Berhe Belay: nega.berhe.belay@mail.com
